# Blue-Light Therapy following Mild Traumatic Brain Injury: Effects on White Matter Water Diffusion in the Brain

**DOI:** 10.3389/fneur.2017.00616

**Published:** 2017-11-22

**Authors:** Sahil Bajaj, John R. Vanuk, Ryan Smith, Natalie S. Dailey, William D. S. Killgore

**Affiliations:** ^1^Social, Cognitive and Affective Neuroscience Laboratory (SCAN Lab), Department of Psychiatry, College of Medicine, University of Arizona, Tucson, AZ, United States

**Keywords:** concussion, diffusion tensor imaging, fractional isotropy, isotropic diffusion, neuropsychological function, quantitative anisotropy, sleep, structural recovery

## Abstract

Mild traumatic brain injury (mTBI) is a common and often inconspicuous wound that is frequently associated with chronic low-grade symptoms and cognitive dysfunction. Previous evidence suggests that daily blue wavelength light therapy may be effective at reducing fatigue and improving sleep in patients recovering from mTBI. However, the effects of light therapy on recovering brain structure remain unexplored. In this study, we analyzed white matter diffusion properties, including generalized fractional anisotropy, and the quantity of water diffusion in isotropic (i.e., isotropic diffusion) and anisotropic fashion (i.e., quantitative anisotropy, QA) for fibers crossing 11 brain areas known to be significantly affected following mTBI. Specifically, we investigated how 6 weeks of daily morning blue light exposure therapy (compared to an amber-light placebo condition) impacted changes in white matter diffusion in individuals with mTBI. We observed a significant impact of the blue light treatment (relative to the placebo) on the amount of water diffusion (QA) for multiple brain areas, including the corpus callosum, anterior corona radiata, and thalamus. Moreover, many of these changes were associated with improvements in sleep latency and delayed memory. These findings suggest that blue wavelength light exposure may serve as one of the potential non-pharmacological treatments for facilitating structural and functional recovery following mTBI; they also support the use of QA as a reliable neuro-biomarker for mTBI therapies.

## Introduction

Mild traumatic brain injury (mTBI) is a common and often unobtrusive wound that occurs when kinetic energy is transferred to the brain through some form of traumatic event, such as a fall, blow to the head, or blast wave. While there are typically no exceptionally conspicuous physical or neuroimaging signs of mTBI, the mechanical trauma to the brain leads to a mild temporary disruption of consciousness or other alteration of ongoing cognition. Also commonly known as “concussion,” mTBI can further lead to persistent alterations in neuropsychological functions, including changes in mood (e.g., depression), poor attention and concentration, and memory problems (1, 2). Importantly, sleep deprivation is also known to produce many of these same symptoms (3, 4). It is therefore possible that sleep disturbances following mTBI may cause, or at least exacerbate, ongoing post-concussion symptoms. However, the nature of these complaints and their contribution to the experience of daytime sleepiness is not well understood (5). An objective measure of daytime sleepiness is the multiple sleep latency test (MSLT), which is used to determine the time it takes an individual to fall asleep (sleep onset latency) when given the opportunity to take a nap. Following a head trauma, symptoms are believed to result from neuronal damage in the form of diffuse axonal injury (6, 7), leading to the release of specific proteins that in turn promote maladaptive functional and structural changes within the brain (8). Identifying neuro-markers of these changes remains an important challenge in ongoing attempts to understand and treat mTBI and post-concussive symptoms.

A very limited number of treatment options for mTBI have been proposed and experimentally validated. Available treatments include cognitive behavior therapy (9), neuropsychological rehabilitation (10), educational intervention (11), and pharmacological intervention (12). Although the effects are small, some intervention studies report reliable reductions in post-concussion symptoms, including sleep problems, following successful treatment (13). Considering a range of post-concussion symptoms can also occur as the result of sleep loss, it is likely that improving sleep quality in particular would also lead to improvements of other post-concussion symptoms, such as attention, concentration, memory, and mood disturbances. While improving sleep makes sense, this is often easier said than done. A natural and potentially powerful method for regulating the sleep–wake cycle is through targeted exposure to bright light in the morning hours. Exposure to short wavelength light (~430–475 nm; blue wavelength light) has been demonstrated as an alternative to pharmacological treatment methods that focus on improving alertness, concentration, daytime sleepiness, as well as sleep quality (14, 15). Intrinsically photosensitive retinal ganglion cells are particularly responsive to light within the short wavelengths. These cells transmit signals to hypothalamic nuclei, which in turn regulate the production of melatonin (16, 17). Morning exposure to blue wavelength light leads to a suppression of melatonin production, which contributes to a phase delay and stabilization of the circadian rhythm (18), increases daytime alertness and vigilance, and earlier onset of evening sleep (19, 20). Interestingly, a recent clinical trial showed that 4 weeks of 45 min of morning blue-light therapy (BLT) in comparison to longer wavelength placebo light was effective at reducing self-rated fatigue and daytime sleepiness among individuals recovering from TBI (21). However, the extent to which these behavioral changes correspond to structural changes within the brain has not been explored.

When considering the potential influences of BLT on mTBI, it is important to consider that mTBI is associated with microscopic changes in brain structure, particularly within the white matter axonal tracts. Abnormalities in fractional anisotropy (FA) in the brain following an mTBI have been studied extensively using diffusion tensor imaging (DTI), a method that allows high-resolution imaging of the directional movement of water molecules along axonal fiber tracts (i.e., how fast water molecules move along fiber tracts). Abnormalities in FA in individuals with an mTBI are reported in areas such as uncinate fasciculus (UF) (22), superior longitudinal fasciculus (SLF) (23), anterior corona radiata (ACR) (22), corpus callosum (CC) (24), and thalamus (25). Alterations in FA within (a) UF are reported to be associated with changes in Mini-Mental State Examination (MMSE) scores (cognitive function) and specifically, memory performance (22, 26); (b) SLF and CC are reported to be associated with executive function (attention and memory) (27); (c) ACR changes are correlated with changes in attention (22); and (d) anterior thalamic nucleus changes are also linked to changes in executive function, memory, and attention (25). In addition, studies have found that individuals with mTBI show alterations in white matter within the frontal lobe (frontal cortex/dorsolateral prefrontal cortex, DLPFC), and that these alterations are correlated with lower executive control and related cognitive functions (28). Also, compared to healthy controls (HCs), there are multiple studies that have reported abnormally high FA values in individuals with mTBI within several areas, including the genu and splenium of CC, ACR (bilaterally), lUF, and internal capsule (IC; bilaterally) (29, 30). Recently, new diffusion measures—quantitative anisotropy (QA), isotropic diffusion (ISO), and generalized fractional anisotropy (GFA)—were introduced to the field of DTI for the analysis of diffusion properties of white matter (31). QA and ISO represent *how much* water diffuses (i.e., density) in a specific/restricted direction and in an isotropic fashion (i.e., total isotropic component), respectively. In contrast, GFA, which is calculated from an orientation distribution function, is a measure of *how fast* water diffuses (i.e., diffusivity) in an anisotropic fashion, i.e., it represents degree to which diffusion is anisotropic (31, 32). Highly significant correlations between FA and GFA were reported in the past (33). In addition, the difference between QA and GFA pertains to the fact that QA is a measure of water diffusion along each fiber orientation, whereas GFA/FA is defined for each voxel. Compared to GFA/FA, QA is also reported to have lower susceptibility to partial volume effects of crossing-fibers, free-water diffusion in ventricles, and non-diffusive particles (31). Moreover, normalization of QA helps to stabilize the spin-density measurement across subjects. In this study, we investigated multiple diffusion measures (i.e., diffusivity as well as density measures) simultaneously to better characterize the white matter properties; therefore, in conjunction with GFA, we also estimated normalized QA (NQA) and ISO measures. To the best of our knowledge, no study to date has used these metrics simultaneously to examine the effect of light exposure treatment on the brain following mTBI.

In individuals with mTBI, how changes in post-concussion symptoms following an exposure to BLT may correspond to structural changes within the brain has not yet been explored. Recent evidence suggests that sleep is important for clearing the neurotoxins that build up throughout the day (34) and increases the production of oligodendrocyte progenitor cells that contribute to myelin formation (35), which could conceivably facilitate repair of axonal damage. Based on this, we hypothesized that 6 weeks of daily morning BLT, compared to a placebo condition with an amber-light therapy (ALT) device, would improve sleep and, consequently, lead to changes in white matter water diffusion, improvements in cognitive abilities such as attention and memory, and daytime sleepiness. To this end, we investigated whether individuals in the BLT and ALT groups showed significant changes in diffusion (i.e., GFA, NQA, and ISO), cognitive, and sleep measures. Furthermore, we examined the correlations between changes in diffusion measures from pre- to post-treatment and changes in neuropsychological performance and sleep onset latency.

## Materials and Methods

### Participants

Twenty-eight individuals meeting criteria for mTBI (mean age = 21.48 ± 3.76 years, 15F) underwent neuroimaging using a Siemens Tim Trio 3T scanner (Erlangen, Germany) at the McLean Hospital Imaging Center. The majority of the individuals (19 out of 28) sustained an mTBI while engaged in a physical activity (e.g., soccer, rugby, hiking, and karate); whereas 9 individuals sustained an mTBI during either a vehicular or household accident. All of the mTBI individuals had a documented mTBI within the preceding 12 months, but not sooner than 4 weeks before their screening. An mTBI was defined based on the criteria established by the VA/DoD practice guidelines (36) as a traumatically induced event (e.g., head impact, blast wave) that was associated with an alteration in mental status (e.g., confusion, disorientation, retrograde, or anterograde amnesia), consciousness (i.e., loss of consciousness less than 30 min; alteration of consciousness up to 24 h), post-traumatic amnesia up to 24 h, and a Glascow Coma Scale from 13 to 15. All participants were right-handed and primary English speakers. All study participants were required to have some level of self-reported sleep problem, e.g., if they were sleepier during the day, having difficulty in sleeping at night and staying alert during the day, etc. Therefore, all participants were screened using a set of sleep questionnaires where they indicated self-reported sleep problems and endorsed that the sleep problems either emerged or worsened following the injury. Participants with any history of neurological, mood, or psychotic disorder with an onset before the mTBI, or who suffered a loss of consciousness exceeding 30 min following an injury were excluded. Participants were thoroughly briefed on the potential risks and benefits of the study and all completed written informed consent before enrollment. Participants were financially compensated for their time. The experimental protocol was approved by the Institutional Review Board of McLean Hospital, Partners Health Care, and the U.S. Army Human Research Protections Office. All procedures performed in this study were conducted in accordance with the ethical standards of the institutional and/or national research committee and with the 1964 Helsinki declaration and its later amendments or comparable ethical standards.

### Protocol

All the participants underwent DTI, neuropsychological testing, and MSLT sessions on two occasions; separated by 6 weeks of daily morning light therapy with either blue light or a sham placebo amber light. Participants were instructed to rest and relax during scanning. All data were collected within a period of 3 years. All eligible participants completed daily sleep diaries and questionnaires and were fitted with a wrist actigraph for sleep monitoring throughout the period of the study. Participants were asked to use a commercially available light therapy device (GoLite Blu^®^, Philips Electronics) for 6 weeks (i.e., 30 min everyday within 2 h of awakening, but before 11:00 a.m.). Half of the individuals (*N* = 14, mean age = 21.75 ± 4.43 years, 8F) were randomly assigned to BLT and half (*N* = 14, mean age = 21.21 ± 3.09 years, 7F) were assigned to ALT. ALT and BLT groups did not differ significantly in age [*F*(1,26) = 0.14, *p* > 0.05, one-way ANOVA], gender [χ^2^(1) = 0.14, *p* > 0.05, Pearson’s Chi-square], and body-mass index [*F*(1,25) = 2.77, *p* > 0.05, one-way ANOVA]. However, two important covariates were included in our analyses: (1) “light compliance” was calculated as the percentage of the total number of days that the participant acknowledged actually using the light *via* self-report divided by the total number of days in the study (i.e., number of days between baseline and post-treatment assessments), and (2) “time since injury,” which was calculated as the number of days between the index mTBI and the baseline assessment.

### DTI Data Acquisition and Image Processing

Diffusion-weighted imaging (DWI) data were acquired along 72 directions with a *b*-value = 1,000 s/mm^2^, voxel size = 1.75 mm × 1.75 mm × 3.5 mm, flip angle = 90°, repetition time (TR) = 6,340 ms, echo time (TE) = 99, slices thickness = 3.5 mm, and number of slices = 40 encompassing the whole brain. A set of eight images with no diffusion weighting (b0 images) was also acquired. Using dcm2nii toolbox [part of MRIcron (37)], we converted DWI data from DICOM into NIFTI format. A *b*-value and *b*-vector file was generated during this step. Next, we performed standard eddy current correction using FMRIB Software Library v6.0 processing software package^1^ on DWI data for head motion correction.

### Neuropsychological Assessments and Sleep Measures

The Repeatable Battery for the Assessment of Neuropsychological Status (RBANS) (38), which included scales assessing delayed memory (DM), immediate memory (IM), attention (ATT), visuospatial/constructional (VC), and language (LAN) abilities was administered pre- and post-light exposure.

The RBANS IM is a measure of initial encoding and learning of simple and complex verbal information and RBANS DM is a measure of delayed recall of visual and verbal stimuli and recognition of verbal stimuli. The RBANS ATT is a measure of speed and accuracy of information processing. The RBANS VC is a measure of visuospatial perception, and RBANS LAN is a measure of ability to express language. Lower RBANS IM and DM scores represent difficulty in the recognition and recall of long-term memories and verbal learning, respectively. Lower RBANS ATT scores represent difficulty in the basic attention processes. Lower RBANS VC and LAN scores represent difficulty with using visuospatial information and language (expressive and receptive), respectively. The use of the RBANS has been shown to be clinically valid and reliable screening tool to assess cognitive deficits following traumatic brain injury (39).

The MSLT has been shown to better reflect the degree of daytime sleepiness when compared to self-report, and with high test–retest reliability (40–42). During each assessment session, participants underwent a modified MSLT protocol, using a standard electrode montage for polysomnographic (PSG) recording (ALICE LE^®^, Phillips Respironics). Signals were recorded from EEG (C3A2, C4A2, O1A1, and O2A2), electrooculogram, submental electromylogram, and electrocardiogram. On three occasions throughout the testing session (11:50 a.m., 1:50 p.m., 3:50 p.m.), participants were given a 20-min opportunity to take a nap in a sound attenuated bedroom. Increased sleep propensity and/or abnormal daytime sleepiness is inferred from decreased sleep onset latency during these MSLT trials. PSG recordings were monitored for the duration and then scored by certified sleep technicians using 30-s epochs and Somnologica software. Sleep onset latency was classified as the first epoch in which >50% was identified as any stage of sleep. Sleep onset latency was quantified for each trial, as well as the average onset latency across the three MSLT administrations.

### Data Analysis

For each participant, we estimated water diffusion parameters such as mean GFA, mean NQA, and mean ISO, using the Q-space diffeormophic reconstruction (QSDR) approach (43) implemented in DSI Studio.^2^ QSDR is a model-free approach, which calculates the density distribution of water diffusion using a high-resolution standard brain atlas constructed from 90-diffusion spectrum imaging datasets in the ICBM-152 space. Tractography was performed using 25,000 sub-voxel seeds in each region of interest for each participant. A turning angle threshold of 60°, QA threshold of 0.10, and length constrained between 30 and 200 mm was used to estimate diffusion parameters. To ensure consistency across subjects, we normalized the QA measure by scaling the subject-wise maximum QA value to 1. Normalization of QA assumes that all the subjects have identical compactness of white matter. In order to avoid any bias among participants, an identical set of tracking parameters was used for each participant before and after the light therapy. For each participant, GFA, NQA, and ISO were estimated for all the possible tracts crossing 11 brain areas, namely the DLPFC, the genu, body and splenium of the CC, the left and the right uncinate fasciculus (lUF and rUF), the left and the right superior longitudinal fasciculus (lSLF and rSLF), the left and the right anterior corona radiata (lACR and rACR), and the thalamus. DLPFC is attributed anatomically to Brodmann areas (BAs) 9 and 46 (44). To define DLPFC in this study, we integrated BAs 9 and 46 whereas we used the ICBM-DTI-81 white matter labels atlas (45) and the JHU white matter tractography atlas (45) (implemented in DSI Studio) to define all other regions of interest. Diffusion parameters (GFA, NQA, and ISO) from tracts crossing the 11 specified seed regions were used in the analyses. In order to estimate the diffusion measures across all the possible tracts crossing each of the 11 brain areas, no waypoint regions of interest were included in the analysis.

Metrics of GFA, NQA, and ISO were compared using mixed analysis of covariance (ANCOVA), with light group (BLT/ALT) as a between groups variable and session (pre- versus post-treatment) as a within-subjects variable, while “time since injury” and “light compliance” were included as nuisance covariates. To test the association between the changes in white matter integrity with changes in neuropsychological performance and sleep latency measures, change metrics for each variable were evaluated with partial correlations, controlling for time since injury and light compliance. For this analysis, we used residualized change scores derived by regressing post-treatment scores on pre-treatment scores and determining the residual value for each participant. This provides a metric of post-treatment status controlling for pre-treatment status (i.e., residualized change). We report false discovery rate (FDR) corrected *p*-values for the partial correlations.

## Results

**Figure 1:**
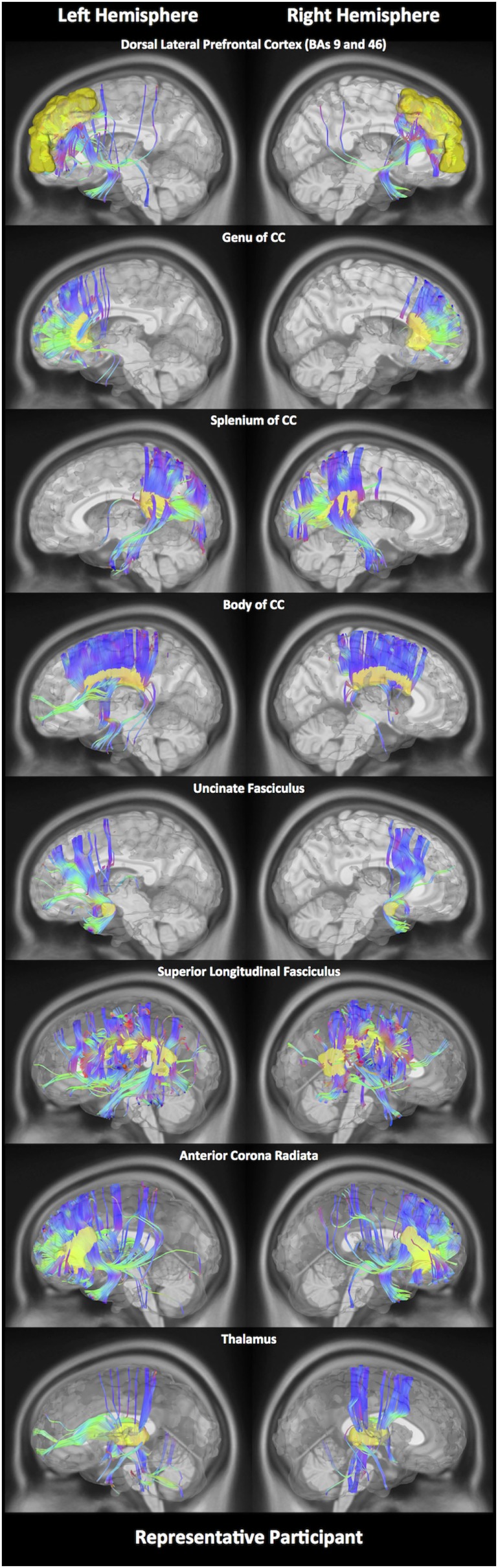
White matter fiber tracking. Here, for a representative participant, we illustrate white matter fiber tracts for each of the 11 regions. Tracts shown in red indicate a fiber direction from left to right or *vice versa*. Blue indicates a fiber direction from anterior to posterior or *vice versa*. Green indicates a fiber direction from superior to inferior or *vice versa*.

In order to estimate different diffusion parameters, we first performed whole-brain tractography, followed by limiting the white matter tracts to those passing through 11 predefined seed regions, namely—R01: the DLPFC, R02: genu, R03: body, R04: splenium of the CC, R05: the lUF, R06: the rUF, R07: the lSLF, R08: the rSLF, R09: the left anterior corona radiata (ACR), R10: right anterior corona radiata (ACR), and R11: the thalamus. Selection of these 11 regions was purely based on previous literature showing abnormalities water diffusion in these regions following mTBI (22–28). In Figure 1, we show fiber tracts crossing through each region for a representative participant. Here, fibers are colored coded to represent their direction, where “red” indicates fibers along the *X*-axis (i.e., left–right), “green” indicates fibers along the *Y*-axis (i.e., anterior–posterior), and “blue” indicates fibers along the *Z*-axis (i.e., inferior–superior).

### Effect of Light Therapy on Diffusion Properties of the Brain following an mTBI

A detailed comparison of diffusion parameters, GFA, NQA, and ISO, was performed on fiber pathways crossing through the 11 specified seed regions (R01 to R11). All the results were corrected for multiple comparisons using Bonferroni’s method. In Figure S1 in Supplementary Material, for each area, we showed subject and fiber averaged GFA, NQA, and ISO measures before and following 6 weeks of either ALT or BLT, where error bars represent the SEM. The presented data in Figure S1 in Supplementary Material are raw data, which are uncorrected for confounds.

#### GFA

**Figure 2:**
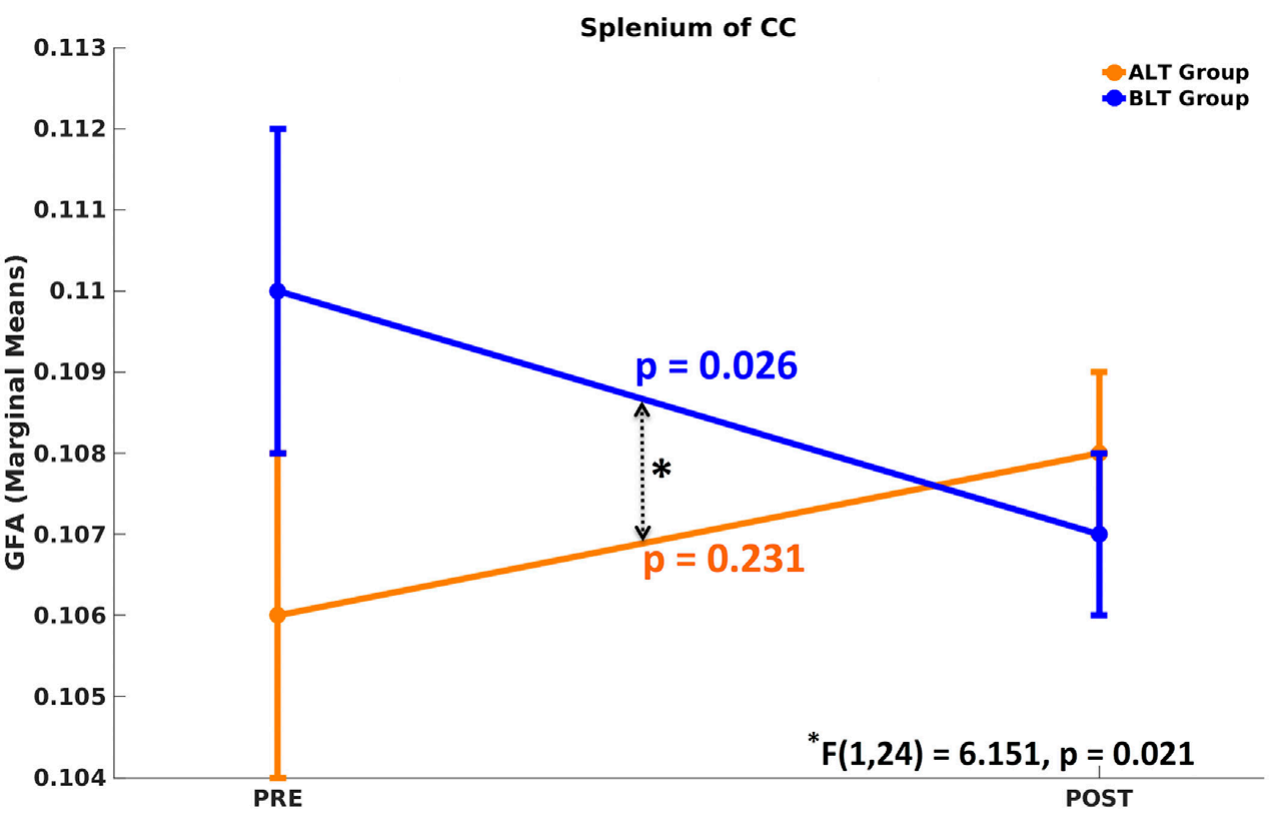
Mixed analysis of covariance of generalized fractional anisotropy (GFA). Compared to baseline, only the fibers crossing the splenium of corpus callosum (CC) showed significant differences in GFA following blue-light therapy (BLT). No significant difference in GFA was found for fibers crossing any brain area, including the splenium of CC, following amber-light therapy (ALT).

There was a significant time (pre- and post-treatment) × group (ALT/BLT) interaction [*F*(1,24) = 6.151, *p* = 0.021] such that following BLT, but not ALT, individuals showed a significant decrease in GFA for only the fibers crossing the splenium of the CC. Furthermore, within-subject pairwise comparison showed a significant decrease in GFA following BLT [*F*(1,24) = 5.619, *p* = 0.026], but not ALT [*F*(1,24) = 1.511, *p* = 0.231]. ANCOVA results for GFA of the fibers crossing the splenium of the CC are summarized in Figure 2 and Table 1.

**Table 1:** Summary of analysis of variance (repeated measures ANOVA) for GFA.

Within-subjects effects					
**Interaction**					

**Source**	**Brain areas**	**Type III sum of squares**	**Mean square**	***F*(1, 24)**	**Significance (GFA)**

Time (pre and post) × group (ALT/BLT) (sphericity assumed)	Splenium of CC	0.000	0.000	6.151	0.021*

**Pairwise comparisons (pre versus post)**					

**Effect of treatment**	**Brain areas**	**Groups**	***F***(1, 24)		**Significance (GFA)**

Pre versus post	Splenium of CC	ALT	1.511		0.231
		BLT	5.619		0.026**

*GFA, generalized fractional anisotropy; BLT, blue-light therapy; ALT, amber-light therapy; CC, corpus callosum*.

*Interaction is significant at p < 0.05.

**Mean difference between post- and pre-treatment is significant at p < 0.05.

#### NQA

**Figure 3:**
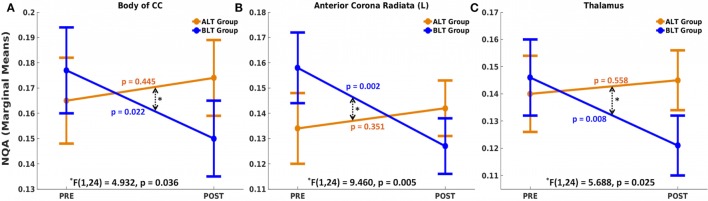
Mixed analysis of covariance of normalized quantitative anisotropy (NQA). Fibers crossing three brain areas, the body of the corpus callosum (CC) **(A)**, left anterior corona radiata (ACR) **(B)**, and thalamus **(C)** showed significant time (pre and post) × group (ALT/BLT) interaction in normalized QA (NQA). Compared to baseline, pairwise comparison showed significant reduction in NQA for these three regions following BLT, but not following ALT. BLT, blue-light therapy; ALT, amber-light therapy.

There was a significant time (pre- and post-treatment) × group (ALT/BLT) interaction such that following BLT, but not ALT, individuals showed a significant decrease in NQA for the fibers crossing three brain areas, i.e., body of CC [*F*(1,24) = 4.932, *p* = 0.036], the left ACR [*F*(1,24) = 9.460, *p* = 0.005], and thalamus [*F*(1,24) = 5.688, *p* = 0.025]. Furthermore, pairwise comparison showed that following BLT, there was significant decrease in NQA for the fibers crossing these three areas, i.e., body of CC [*F*(1,24) = 5.984, *p* = 0.022], the left ACR [*F*(1,24) = 12.347, *p* = 0.002], and thalamus [*F*(1,24) = 8.226, *p* = 0.008], but not following ALT. ANCOVA results for NQA of the fibers crossing these three areas are summarized in Figure 3 and Table 2.

**Table 2:** Summary of analysis of variance (repeated measures ANOVA) for normalized quantitative anisotropy (NQA).

Within-subjects effects (ANCOVA)					
**Interaction**					

**Source**	**Brain areas**	**Type III sum of squares**	**Mean square**	***F***(1, 24)	**Significance^a^ (NQA)**

Time (pre and post) × group (ALT/BLT) (sphericity assumed)	Body of CC	0.004	0.004	4.932	0.036*
	Left anterior corona radiata	0.005	0.005	9.460	0.005*
	Thalamus	0.003	0.003	5.688	0.025*

**Pairwise comparisons (pre versus post)**					

**Effect of treatment**	**Brain areas**	**Groups**	***F***(1, 24)		**Significance^a^ (NQA)**

Pre versus post	Body of CC	ALT	0.604		0.445
		BLT	5.984		0.022**

	Left anterior corona radiata	ALT	0.903		0.351
		BLT	12.347		0.002**

	Thalamus	ALT	0.352		0.558
		BLT	8.226		0.008**

*NQA, normalized quantitative anisotropy; BLT, blue-light therapy; ALT, amber-light therapy; CC, corpus callosum; ANCOVA, analysis of covariance*.

*^a^Adjustment for multiple comparisons using Bonferroni’s method*.

*Interaction is significant at p < 0.05.

**Mean difference between post- and pre-treatment is significant at p < 0.05.

#### ISO

There were no significant changes in ISO for fibers crossing any of the 11 areas from pre- to post-treatment for either group.

### Effect of Light Therapy on Neuropsychological Function and Sleep Onset Latency

Contrary to our expectations, we did not find significant time (pre- versus post-treatment) × group (ALT/BLT) interaction for neuropsychological function and sleep onset latency. However, because we found significant differences in GFA (for 1 out of 11 brain areas) as well as in NQA (for 3 out of 11 brain areas) following BLT, we then examined whether individual differences in white matter within these 4 brain regions were related to individual differences in our behavioral measures of neuropsychological function (attention and memory) and daytime sleep onset latency during the MSLT trials. Specifically, partial regression analyses were performed (corrected for “time since injury” and “light compliance”) between diffusion measures (GFA and NQA) and neuropsychological function measures (i.e., RBANS scores) as well as sleep onset latency.

#### Neuropsychological Function

**Figure 4:**
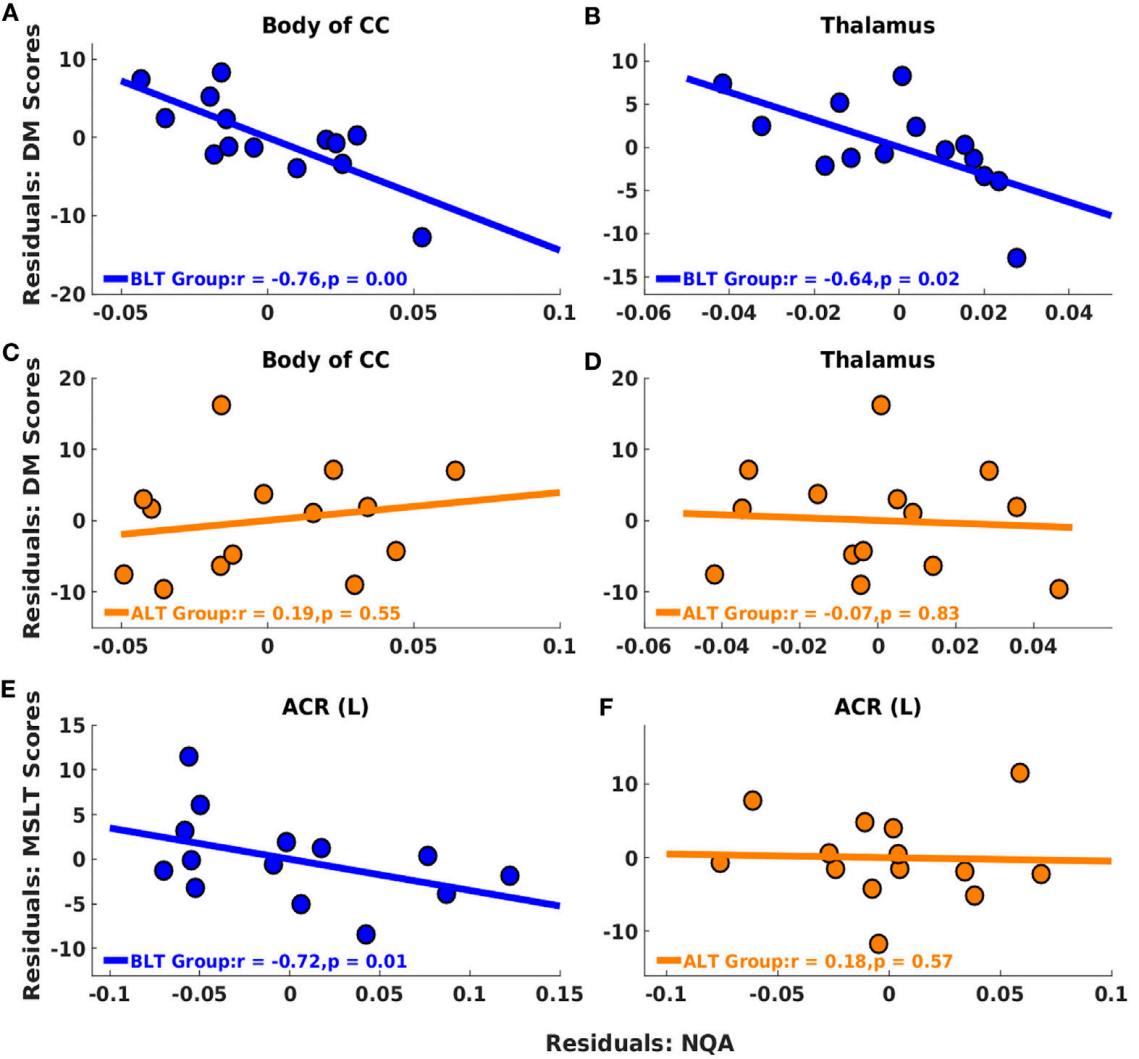
Associations between residualized changes in white matter diffusion measures, neuropsychological measures, and multiple sleep latency test (MSLT) scores following BLT and ALT. For BLT and ALT groups, correlations found between residualized changes in NQA and neuropsychological function measures (DM) [BLT: **(A,B)**, ALT: **(C,D)**], and between residualized changes in NQA and MSLT scores [BLT: **(E)**, ALT: **(F)**] are reported. Significant negative correlations between residualized changes in Repeatable Battery for the Assessment of Neuropsychological Status DM scores and NQA measures were found for fibers crossing the body of the corpus callosum (CC) **(A)** and thalamus **(B)** for BLT group. No significant correlations were found for ALT group, including for fibers crossing the body of the CC **(C)** and thalamus **(D)**. Significant negative correlations between residualized changes in sleep onset latency during the first MSLT administration and NQA measures were also found for fibers crossing the left anterior corona radiata (ACR) **(E)** for BLT group but not for ALT group **(F)**. BLT, blue-light therapy; ALT, amber-light therapy, DM, delayed memory.

Following BLT or ALT, we did not find significant association between residualized changes in any neuropsychological measures or MSLT scores and residualized changes in GFA for fibers crossing the splenium of the CC. But significant negative partial correlations were observed between residualized changes in RBANS DM scores and residualized changes in NQA for fibers crossing two brain areas: the body of the CC (*r* = −0.76, *p* = 0.00; FDR corrected *p* = 0.02) (Figure 4A) and the thalamus (*r* = −0.64, *p* = 0.02; FDR corrected *p* = 0.02) (Figure 4B), i.e., greater changes in NQA were associated with better DM performance following BLT. After multiple comparisons correction, we did not find significant association between residualized changes in any neuropsychological measure and residualized changes in NQA for fibers crossing any of the regions of interest following ALT (Figures 4C,D).

#### Daytime Sleep Onset Latency

Significant negative partial correlations were observed between residualized changes in sleep onset latency during the first MSLT administration and residualized changes in NQA for fibers crossing ACR (L) (*r* = −0.72, *p* = 0.01; FDR corrected *p* = 0.01) (Figure 4E), i.e., greater changes in NQA were associated with delayed sleep onset latency during the day following BLT. However, after multiple comparisons correction, we did not find significant association between residualized changes in sleep onset latency during any of the MSLT administrations and residualized changes in NQA for fibers crossing any of the regions of interest following ALT (Figure 4F). The findings above are summarized in Table 3.

**Table 3:** Summary of correlations between residualized changes in neuropsychological function measures (DM) and GFA, and between residualized changes in MSLT scores and normalized quantitative anisotropy (NQA) measures.

#	ROIs	Partial correlations (*r*, *p*) between residualized changes in									
		GFA and					NQA and				
		DM	MSLT 1	MSLT 2	MSLT 3	Mean MSLT	DM	MSLT 1	MSLT 2	MSLT 3	Mean MSLT
**BLT group**											

1	Splenium of CC	0.35, 0.27	−0.33, 0.29	−0.17, 0.60	−0.48, 0.12	−0.37, 0.23	–				
2	Body of CC	–					−0.76, 0.00**	−0.18, 0.58	−0.43, 0.16	−0.17, 0.60	−0.37, 0.24
3	ACR (L)	–					−0.37, 0.23	−0.72, 0.01**	−0.45, 0.14	−0.45, 0.15	−0.58, 0.05
4	Thalamus	–					−0.64, 0.02**	−0.45, 0.14	−0.48, 0.11	−0.39, 0.21	−0.54, 0.07

**ALT group**											

1	Splenium of CC	−0.02, 0.96	−0.56, 0.06	−0.61, 0.04*	−0.38, 0.22	−0.61, 0.04*	–				
2	Body of CC	–					0.19, 0.55	−0.52, 0.08	−0.52, 0.08	−0.51, 0.09	0.60, 0.04*
3	ACR (L)	–					0.43, 0.16	0.18, 0.57	0.04, 0.91	−0.04, 0.92	0.07, 0.83
4	Thalamus	–					−0.07, 0.83	0.13, 0.70	−0.19, 0.56	0.42, 0.17	0.17, 0.60

*ROIs, regions of interest; GFA, generalized fractional anisotropy; NQA, normalized quantitative anisotropy; DM, delayed memory; MSLT, multiple sleep latency test; BLT, blue-light therapy; CC, corpus callosum; ACR (L), anterior corona radiata (left); ALT, amber-light therapy; FDR, false discovery rate*.

*p < 0.05 (uncorrected for multiple comparisons).

**p < 0.05 (FDR corrected for multiple comparisons).

## Discussion

In this study, we analyzed several white matter water diffusion properties including GFA, NQA, and ISO, for fibers crossing several brain areas in individuals with a recent mTBI. From a group of individuals with mTBI, half were randomly assigned to a placebo condition of ALT and the other half to an active condition of BLT. Consistent with our hypotheses, we observed significant changes in some of these white matter properties (i.e., GFA and NQA) for multiple brain areas following BLT. Contrary to our hypotheses, we did not observe significant changes in cognitive abilities such as attention and memory, or in the daytime sleep onset latency measures. However, an analysis of cognitive abilities and daytime sleep onset latency measures in relation to white matter properties revealed a significant relationship between increased DM scores and decreased normalized quantitative anisotropy, as well as an association between increased daytime sleep onset latency and decreased normalized quantitative anisotropy after BLT. These findings suggest that BLT may provide an effective method for facilitating recovery from mTBI.

Previous DTI studies of mTBI have tended to focus on FA as a measure of the diffusion properties of white matter tracts. However, these studies have yielded somewhat inconsistent results, as some report abnormally high (30) and others report abnormally low FA (24) values following an mTBI (i.e., as compared to HCs). Such inconsistencies may be due to several factors, including type, severity and location of injury, time since injury, and variability across subject samples (30). In this study, we examined NQA and ISO, in addition to FA, in order to fully characterize potential treatment effects. In doing so, we found a significant effect of BLT on white matter water diffusion properties (i.e., both GFA and NQA) for several brain areas, which were associated with significant correlations between diffusion measures, behavioral measures of neuropsychological function, as well as daytime sleep onset latency. In contrast, none of the 11 brain areas showed significant change in GFA, NQA, or ISO following the placebo ALT. More specifically, we found that, following BLT (but not ALT), there was significant decrease in GFA for fibers passing through the splenium of the CC, and a significant decrease in NQA for fibers passing through the body of CC, left ACR, and thalamus. These changes in NQA for fiber pathways going through the body of the CC and thalamus were also significantly negatively correlated with changes in RBANS DM scores, suggesting that decreases in NQA were associated with improvements in DM performance from pre- to post-treatment. In addition, changes in NQA for fiber pathways going through the left ACR were significantly negatively correlated with changes in MSLT scores, suggesting that decreases in NQA were associated with improvements in sleep onset latency during the day from pre- to post-treatment. The role of the CC during recovery following BLT might be due to the fact that CC tracts facilitate communication of somatosensory information between parietal and occipital lobes (46) as well as communication between the two cortical hemispheres more generally (47). These tracts are known for their vital role in regulating several advanced brain skills such as memory, learning, and abstract thinking. Damage to these tracts could lead to loss of inter-hemispheric connections, causing multiple neuropsychological impairments (47). Moreover, the CC is also a common site affected following a brain injury. In the previous work, axons of the CC were reported to exhibit multiple stages of degeneration following a traumatic brain injury (48). In addition, generation of myelin sheaths within the CC could be responsible for its greater responsiveness to BLT. Furthermore, the connections between the anterior thalamus and hippocampal gyrus are believed to operate in parallel but with different organization and any damage to such network or any of the participating areas could contribute to impaired memory and discrimination skills (49, 50). The improvements in memory scores as a result of changes in NQA could be due to the effects of blue light exposure on sleep quality, which plausibly results in decreased daytime sleepiness and increases in alertness. Future work assessing PSG or actigraphic changes in sleep duration and quality will be necessary to test these hypotheses directly. In a diffusion kurtosis imaging study, several brain areas including the CC, thalamus, and IC showed correlations between changes in mean kurtosis or radial kurtosis between 1 and 6 months post mTBI and improvements in cognition between the 1- and 6-month visits (51). In that study, no significant differences in other diffusion parameters (such as FA and mean diffusivity) were observed between mTBI patients and age-matched controls. The findings reported in our study are also consistent with previous findings demonstrating microstructural white matter changes in the ACR for patients suffering from narcolepsy, a disorder characterized by rapid sleep onset latency during the daytime (52).

We observed a significant reduction in diffusion measures (GFA and NQA) following BLT. One of the potential explanations for changes in GFA and NQA measures could be attributed to the way axons are packed. Previously, changes in FA are reported to be dependent on axonal packing. It was reported that light axonal packing leaves more intercellular water as compared to dense packing causing less restriction to water molecules, which further results into lower FA values whereas higher degree of myelination results into higher FA values due to tight axonal packing (53). In addition, in a separate study, we recently demonstrated that acute exposure to 30 min of blue light subsequently led to increased functional brain responses within the prefrontal cortex and improved cognitive performance during a working memory task (54). Blue light exposure in the morning may therefore facilitate brain function later during the day, possibly when individuals are at work. If individuals are exposed to 30 min of morning blue light every day for 6 weeks and are able to sustain regular attentional focus, this may plausibly also be reflected in better white matter integrity and improved performance on neuropsychological tasks and decreased daytime sleepiness. Another possible reason for changes in diffusion measures (GFA and NQA) following BLT could be that before BLT, GFA, and NQA were higher and BLT helped to restore these diffusion levels back to normal. In fact, increased water diffusion after an mTBI has been associated with the stretching and deformations of axons following mTBI, which leads to an increase in intra- but decrease in extra-cellular water causing an increase in diffusion along the axons (55, 56). Modeling studies have shown that the inter-hemispheric fibers, especially of the CC, could be more sensitive to mechanical strain following brain deformation after a concussion (57). Diffusion of water molecules through strained axons could further be responsible for higher GFA or NQA. Abnormal disruption of water due to axonal swelling, compression of axons, and expansion of tissues may also lead to abnormal changes in water diffusion (30, 58). Myelin also plays a significant role in axon susceptibility following an mTBI. For instance, compared to myelinated axons, unmyelinated fibers within white matter are more adversely affected following traumatic axonal injury (59). BLT may improve myelination and help in regenerating new structural fibers, which could cause the observed improvements in neurobehavioral scores and possibly the observed changes in GFA and NQA values. However, the potential mechanism behind increased myelination or regeneration of structural fibers following light therapy is not completely understood. It may involve clearance of neurotoxins (34) and increases in oligodendrocyte precursor cells (35) due to shifts in circadian rhythms and improved sleep (18, 60, 61). Previously, it was reported that mean water diffusivity values were reduced within several brain regions including CC, corona radiata, and thalamic radiation in patients with obstructive sleep apnea compared to HCs (62). In a study of patients with bipolar disorder, reduced water diffusivity within the same regions identified here (CC, corona radiata, and thalamic radiation) indicated that sleep quantity could be associated with integrity of myelin sheaths (63). Therefore, BLT may enrich or stimulate the production of myelin-enriched brain debris, which may further stimulate microglial/macrophage activation in white matter tracts (64), especially within the CC, corona radiata, and thalamic radiation, which are associated with various sleep problems. Adaptive alterations in water diffusivity following BLT may also act to strengthen brain function. The association between sleep and variation in diffused water quantity could also be responsible for circadian changes in diffusion measures (65, 66), which may further lead to improvements in brain structure and function following BLT. Furthermore, it is known from other studies that acute exposure to blue light also has a positive impact on brain function and cognitive performance and it makes people faster at responding during working memory tasks without loss of accuracy (54). Separate from the effects of blue light on melatonin suppression, it is possible that blue light may have more direct cognitive alerting effects *via* direct stimulation of the locus coeruleus, which in turn releases norepinephrine throughout the cerebral cortex (54, 67, 68). While speculative, it is conceivable that the effects could be even more robust during periods of insufficient sleep that are extremely common following a traumatic brain injury (69). This is an important area for further research.

Finally, it is noteworthy that NQA appeared to yield a larger number of significant findings than GFA or ISO. One possibility is that NQA is a more sensitive measure to detect microstructural changes of white matter integrity following an mTBI. By contrast, we predict that GFA could be a more sensitive measure to determine white matter differences between controls and mTBI patients. This is also consistent with the previous literature, which has suggested that density measures like NQA are more sensitive to individual physiological differences, whereas diffusivity measures like GFA are more sensitive to pathological conditions (70). NQA is also generally considered to be a more robust measure for deterministic tractography, due to its lower susceptibility to partial volume effects (31). It has also been found that NQA has the capability to filter out noisy fiber tracts, which further results in a higher spatial resolution in NQA-aided tractography. By contrast, voxel-based indices, such as GFA, are not capable of filtering out the noisy fibers since the same magnitude of anisotropy is shared by all the fiber orientations within a voxel (31). These considerations support the idea that NQA-aided tractography may be a better approach than GFA-based tractography for examining abnormal white matter content following an injury and injury-related therapies. It should be noted that the deterministic tractography methods implemented in DSI Studio has achieved the highest “valid connection” examined by an open competition among 96 methods submitted from 20 different research groups around the world.^3^

This study had several limitations. First, we acknowledge the fact that there is no way to assert the accuracy of tractography. Thus, further research will be needed to provide convergent validity to these findings. Second, our data sample was focused on participants with mTBI and did not include healthy controls. The goal was to compare the active versus a placebo condition on the recovery of a patient population, but future work would benefit from a sample of healthy individuals to determine the extent to which the outcomes represent full normalization of brain structure. Third, our mTBI sample also included individuals with different injury mechanisms. Mild injuries of this type are extremely heterogeneous and may vary significantly among samples. Finally, our data sample was relatively small. Low statistical power due to smaller sample size could account for the non-significant findings observed in many neuropsychological function and sleep onset latency measures following BLT.

In summary, these findings provide preliminary evidence that BLT can affect recovery of brain structure and function following mTBI. Following BLT, normalized values of water diffusion were associated with increases in memory and sleep latency scores. While more research is warranted, these preliminary findings raise the possibility that BLT might be useful as a means of facilitating brain and cognitive recovery among individuals with mTBI. Finally, our results also support the use of NQA as a sensitive measure to analyze the effect of treatment following a brain injury.

## Ethics Statement

Participants were thoroughly briefed on the potential risks and benefits of the study and all completed written informed consent before enrollment. The experimental protocol was approved by the Institutional Review Board of McLean Hospital, Partners Health Care, and the U.S. Army Human Research Protections Office (HRPO). All procedures performed in this study were conducted in accordance with the ethical standards of the institutional and/or national research committee and with the 1964 Helsinki declaration and its later amendments or comparable ethical standards.

## Author Contributions

SB conducted the neuroimaging analyses and wrote the initial draft of the manuscript and organized the revisions. JV, RS, and ND each contributed to the writing of revisions of the manuscript and helped with data analysis. WK designed the study, oversaw data collection and analysis, and contributed to writing revisions of the manuscript.

## Conflict of Interest Statement

The authors declare that the research was conducted in the absence of any commercial or financial relationships that could be construed as a potential conflict of interest.

## Footnotes

^1^http://www.fmrib.ox.ac.uk/fsl.

^2^http://dsi-studio.labsolver.org.

^3^http://www.tractometer.org/ismrm_2015_challenge/results.

## Supplementary Material

Figure S1 Subject-averaged generalized fractional anisotropy (GFA), NQA, and isotropic diffusion (ISO) measure. Here, we plot the subject-averaged magnitude of raw diffusion measures before and after either amber-light therapy (ALT) **(A–C)** or blue-light therapy (BLT) **(D–F)** for GFA **(A,D)**, NQA **(B,E)**, and ISO **(C,F)**. Error bars represent the SEM.
