# Supplementary figures and images for: Blue-Light Therapy following Mild Traumatic Brain Injury: Effects on White Matter Water Diffusion in the Brain
Source: Front Neurol. 2017 Nov 22;8:616. doi: 10.3389/fneur.2017.00616 (PMC5702646; doi:10.3389/fneur.2017.00616)

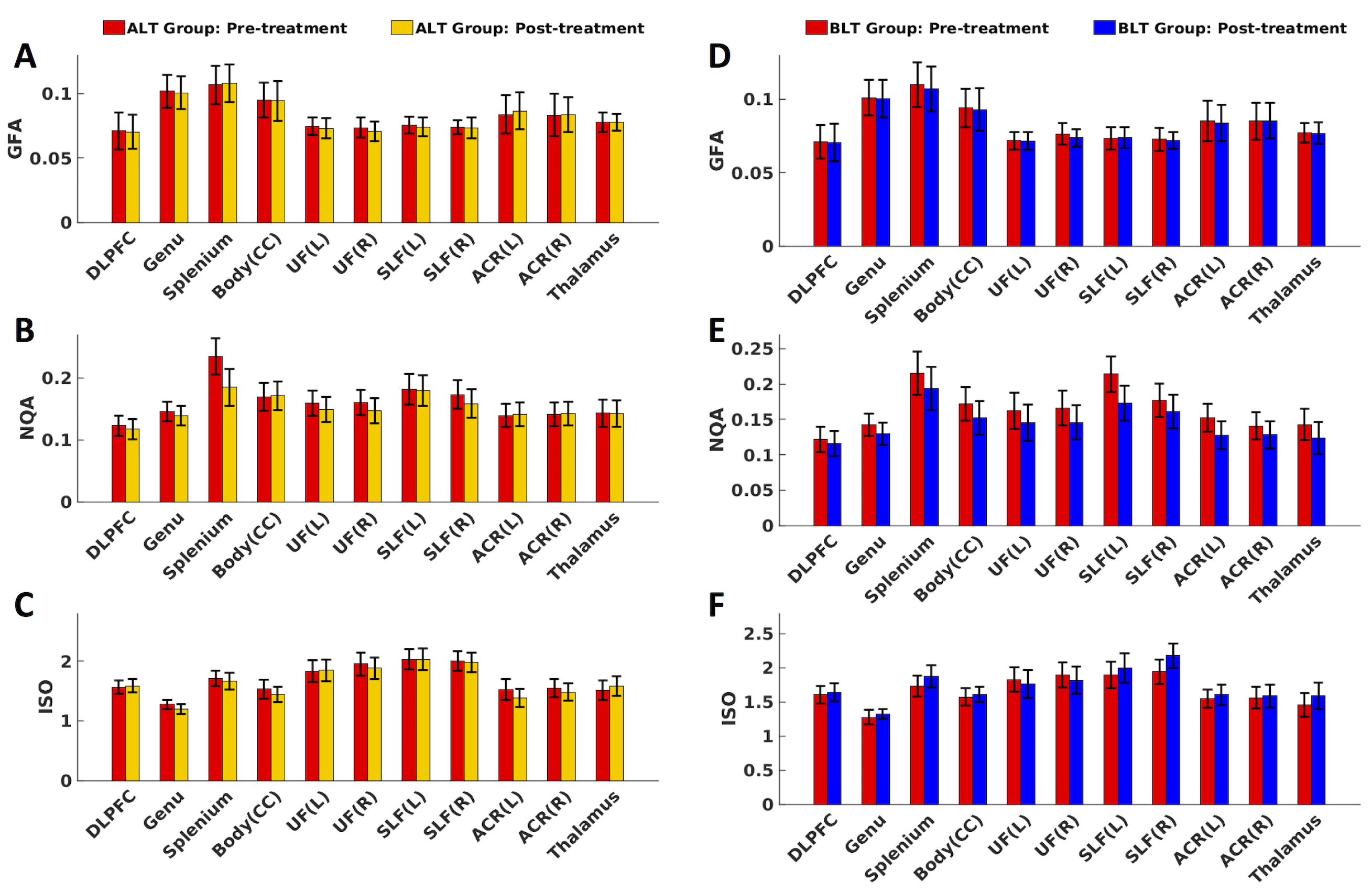

Supplement: Figure S1 — Subject-averaged generalized fractional anisotropy (GFA), NQA, and isotropic diffusion (ISO) measure. Here, we plot the subject-averaged magnitude of raw diffusion measures before and after either amber-light therapy (ALT) (A–C) or blue-light therapy (BLT) (D–F) for GFA (A,D), NQA (B,E), and ISO (C,F). Error bars represent the SEM. [file Image_1.TIFF]
